# Behavioral Consequences of NMDA Antagonist-Induced Neuroapoptosis in the Infant Mouse Brain

**DOI:** 10.1371/journal.pone.0011374

**Published:** 2010-06-29

**Authors:** Carla M. Yuede, David F. Wozniak, Catherine E. Creeley, George T. Taylor, John W. Olney, Nuri B. Farber

**Affiliations:** 1 Department of Psychiatry, Washington University School of Medicine, St. Louis, Missouri, United States of America; 2 Department of Psychology, University of Missouri, St. Louis, Missouri, United States of America; Medical College of Georgia, United States of America

## Abstract

**Background:**

Exposure to NMDA glutamate antagonists during the brain growth spurt period causes widespread neuroapoptosis in the rodent brain. This period in rodents occurs during the first two weeks after birth, and corresponds to the third trimester of pregnancy and several years after birth in humans. The developing human brain may be exposed to NMDA antagonists through drug-abusing mothers or through anesthesia.

**Methodology/Principal Findings:**

We evaluated the long-term neurobehavioral effects of mice exposed to a single dose of the NMDA antagonist, phencyclidine (PCP), or saline, on postnatal day 2 (P2) or P7, or on both P2 and P7. PCP treatment on P2 + P7 caused more severe cognitive impairments than either single treatment. Histological examination of acute neuroapoptosis resulting from exposure to PCP indicated that the regional pattern of degeneration induced by PCP in P2 pups was different from that in P7 pups. The extent of damage when evaluated quantitatively on P7 was greater for pups previously treated on P2 compared to pups treated only on P7.

**Conclusions:**

These findings signify that PCP induces different patterns of neuroapoptosis depending on the developmental age at the time of exposure, and that exposure at two separate developmental ages causes more severe neuropathological and neurobehavioral consequences than a single treatment.

## Introduction

Synaptogenesis, which occurs during the first two weeks after birth in rodents, is a developmental period of heightened sensitivity to certain classes of drugs capable of inducing widespread neuroapoptosis throughout the brain. N-methy-D-aspartate (NMDA) glutamate receptor antagonists (*e.g.* ketamine, phencyclidine [PCP], nitrous oxide) and GABAmimetics (*e.g.* midazolam, phenobarbital, isoflurane) are two classes of agents that produce apoptosis during this vulnerability period [1]–[4]. While there is some overlap in the neuronal populations killed by NMDA antagonists and GABAmimetics, each drug class produces its own distinct damage pattern. Ethanol, possessing both GABAmimetic and NMDA antagonistic properties, also triggers neuroapoptosis in the developing rodent brain [5], which appears to be a composite of the damage patterns resulting from exposure to NMDA antagonists and GABAmimetics. This suggests that both neurotransmitter systems are involved in ethanol-induced apoptotic neurodegeneration.

In humans the synaptogenesis period encompasses the last trimester of gestation and the first few years of life [6]. Because pregnant women or infants are exposed to these classes of agents in clinical and drug abuse settings, there is the potential that these drugs may produce long-lasting functional deficits in vulnerable fetuses and infants. To date, we have conducted two studies to determine whether apoptotic neurodegeneration produced during this period of vulnerability results in measurable cognitive impairments. Specifically, rats treated on postnatal day 7 (P7) with a “cocktail” containing both NMDA antagonists and GABAmimetics (nitrous oxide, isoflurane and midazolam), similar to that used in human obstetric and pediatric anesthesia, show spatial learning and memory deficits when tested as juveniles and as adults [3]. Consistent with these results, mice treated with ethanol on P7 have impairments in spatial learning and memory when tested as juveniles but show some recovery of function by adulthood [7]. The relative contributions of NMDA antagonism and augmentation of GABAergic activity to the cognitive deficits in ethanol-treated mice are currently unknown. Other studies examining effects of multiple doses of PCP (P7, 9 and 11) have found long-term deficits in sensorimotor gating, delayed alternation and adult locomotor activity in rats [8]–[10] and impairments in spatial working memory and social interaction in mice [11]. Therefore, one goal of the conducted research was to determine what long-term behavioral functions would be disrupted in mice with a single exposure on P7 to PCP.

Another important determinant of neuroapoptosis in rodents is a varying sensitivity of different neuronal populations to the apoptogenic effects of NMDA antagonists and GABAmimetic agents, which is highly dependent upon age of exposure. For example, the hippocampus, subiculum, and caudate/putamen are more sensitive during the early periods of synaptogenesis, while other areas, particularly cortical regions and some thalamic nuclei tend to be sensitive in mid to later periods [5]. To date, reports on the long-term behavioral effects of postnatal PCP exposure have focused on P7 or later, which corresponds to the mid to late period of synaptogenesis. Thus, another goal of our research was to determine if exposure to PCP on P2 in mice results in a different pattern of cognitive and behavioral disturbances than those resulting from exposure on P7. In addition, since the developing human brain may be subjected to multiple PCP exposures during synaptogenesis, we also studied the potential long-term functional deficits of mice exposed to PCP during the two periods of vulnerability (P2 and P7).

## Methods

### Ethics Statement

All experimental protocols were approved by the Animal Studies Committee of Washington University in St. Louis in accordance with NIH guidelines.

### Animals

C57BL/6 mice, purchased from Harlan (Indianapolis, IN), were used and given free access to food and water while maintained on a 12 hr on/off light-dark schedule in a temperature- and humidity-controlled room throughout the experiments. Pregnant dams were individually housed in polypropylene cages measuring 30×18.5×13 cm, while offspring were weaned on P21 and group-housed with 5 same-sexed cagemates. Weights and general appearance of the animals were carefully monitored throughout the experiments.

### Pharmacological Agents and Drug Treatments

Phencyclidine (PCP) (Sigma Laboratories, St. Louis, MO) was solubilized in sterile saline diluents for subcutaneous injection according to body weight (10µl/g). Doses used on P2 (35 mg/kg) and P7 (50 mg/kg) were based on results from pilot studies showing that these were doses that produce a robust neuroapoptotic response without causing high levels of mortality at these ages. Animals were randomly assigned to one of six treatment groups: 35 mg/kg PCP on P2; 50 mg/kg PCP on P7; or 35 mg/kg on P2 followed by 50 mg/kg on P7, or matched saline controls. For the histological studies, each of the six groups included 6 animals from 6 different litters to control for litter effects (N = 36).

### Histological Procedures

Immunocytochemical techniques were used to detect neuroapoptosis through activated caspase-3 (AC3) staining as previously described [12]. Specifically, 10 hr after drug exposure for a given PCP or saline control treatment, mice were deeply anesthetized with sodium pentobarbital and perfused with a heparinized saline flush followed by 4% paraformaldehyde in a 0.1 M sodium phosphate buffer. Immediately following perfusion, the whole brain was removed from each animal and post-fixed in perfusate for 4 d. Whole brains were cut into 70 µm sections on a Vibratome and stored in phosphate buffered saline (PBS). Sections were washed in 0.01 M PBS, quenched for 10 min in methanol containing 3% hydrogen peroxide, and then incubated for 1 hr in blocking solution (2% BSA/0.2% milk/0.1% Triton-X 100 in PBS), followed by incubation overnight in rabbit anti-active caspase-3 antiserum (Cell Signaling Technology, Beverly, MA). Sections were incubated for 1 hr in secondary antiserum (goat anti-rabbit 1∶200 in blocking solution), and then reacted in the dark with ABC reagents (standard Vectastain ABC Elite Kit; Vector Laboratories, Burlingame, CA) for 1 hr. The sections were then washed 3 times with PBS, and incubated with VIP reagent (Vector VIP substrate kit for peroxidase, Vector Labs, Burlingame, CA). To quantify differences in neuroapoptosis between P7 and P2+P7 treatments, three coronal sections were selected from each animal, one including the caudate and anterior cingulate cortex (P6 #18, 3.51 mm), laterodorsal thalamic nuclei (P6 # 27, 4.59 mm) and retrosplenial cortex (P6 #33, 5.31 mm), [13]. Caspase-postive neurons in the cortex, caudate, and thalamus were counted using StereoInvestigator 7 (MicroBrightfield, Inc., Williston, VT) and a mean density score was derived for each animal.

### General Design of Behavioral Experiments

In the first behavioral experiment, a total of 32 mice were used from 5 litters. One male and one female pup from each litter received an injection of 50 mg/kg of PCP on P7. Litter-matched controls received saline injections. All pups were placed in a warm (30°) environment resembling the home nest until the effects of the drug appeared to dissipate (10 hr), and then returned to their respective dams. In two subsequent experiments mice were exposed to PCP or saline on P2 (N = 60) or P2+P7 (N = 60). Both genders were represented in each group, and litter-matching techniques were used to assign groups. A second saline control group was included in these experiments to assess the effects of maternal deprivation alone at these time points. Pups from each litter were therefore assigned to one of 3 groups (PCP, saline, or saline+maternal deprivation) such that no treatment or control condition was repeated within the same litter. Mice exposed to PCP or saline in all three experiments (P2, P7, and P2+P7) were evaluated behaviorally during the juvenile period and into adulthood on the following measures: (1) on P23-24, 1 hr locomotor activity and a battery of sensorimotor tests (walking initiation, ledge, inclined and inverted screens and pole, described in further detail in [7]); (2) the Morris water navigation test to evaluate spatial learning and memory at P28, P75, and P170; (3) acoustic startle and pre-pulse inhibition at P50; and (4) Pavlovian fear conditioning on P60.

### Spatial Learning and Memory

Spatial learning and memory were evaluated in the mice using the Morris water navigation test which involved training mice to locate a platform in a pool of opaque water using methods similar to those previously described [7]. The procedure included cued trials (visible platform, varied location, 4 trials/day, 2 days) conducted on P28–29, followed by place trials (submerged platform, fixed location, 2 trials/day, 10 days) beginning on P30. Release points each day were pseudorandomly determined across the four pool quadrants such that each release point was used equally. Probe trials (platform removed) were conducted 1 hr after the last place trial on days 5 and 10. Mice were re-evaluated on the place condition on P75 (±2 days) using the same procedures except that a different submerged platform location was used in the presence of different extramaze cues. Probe trials on days 5 and 10 of place training were also different in that mice received a 30 s probe before testing on day 5 or 10 (24 hr delay) and a 30 s probe after place training on those days (1 hr delay). Mice in the P2+P7 experiment were also tested on cued, place and probe conditions on P170 using a different submerged platform location and different extramaze cues.

### Acoustic Startle Response (ASR) and Pre-Pulse Inhibition (PPI)

The ASR to a 120 dBA auditory stimulus pulse (40 ms broadband burst) and PPI (response to a prepulse plus the startle pulse) were measured concurrently in the mice on P50 using previously described methods [14], [15]. Beginning at stimulus onset, 65, 1 ms force readings were averaged to obtain an animal's startle amplitude. A total of 20 startle trials were presented over a 20 min test period during which the first 5 min served as an acclimation period when no stimuli above the 65 dB white noise background were presented. The session began and ended by presenting 5 consecutive startle (120 db pulse alone) trials unaccompanied by other trial types. The middle 10 startle trials were interspersed with PPI trials (consisting of an additional 30 presentations of 120 dB startle stimuli preceded by pre-pulse stimuli of either 4, 12, or 20 dB above background (10 trials for each PPI trial type). A percent PPI score for each trial was calculated using the following equation: %PPI = 100*(ASRstartle pulse alone - ASRprepulse+startle pulse)/ASRstartle pulse alone (see [14] for greater details).

### Conditioned Fear

A previously described protocol [16] was used to train and test mice using two clear-plastic conditioning chambers (26×18×18 cm high) (Med-Associates, St. Albans, VT) which were easily distinguished by different olfactory, visual, and tactile cues present in each chamber. On day 1, each mouse was placed into the conditioning chamber for 5 min and freezing behavior was quantified during a 2 min baseline period. Freezing (no movement except that associated with respiration) was quantified using *FreezeFrame* image analysis software (Actimetrics, Evanston, IL) which allows for simultaneous visualization of behavior while adjusting for a “freezing threshold” during 0.75 s intervals. After baseline measurements, a conditioned stimulus (CS) consisting of an 80 dB tone (white noise) was presented for 20 sec followed by an unconditioned stimulus (US) consisting of a 1 s, 1.0 mA continuous foot shock. This tone-shock (T/S) pairing was repeated each minute over the next 2 min, and freezing was quantified after each of the three tone-shock pairings. Twenty-four hours after training, each mouse was placed back into the original conditioning chamber to test for fear conditioning to the contextual cues in the chamber. This involved quantifying freezing over an 8 min period without the tone or shock being present. Twenty-four hours later, the mice were evaluated on the auditory cue component of the conditioned fear procedure, which included placing each mouse into the other chamber containing distinctly different cues. Freezing was quantified during a 2 min “altered context” baseline period as well as over a subsequent 8 min period during which the auditory cue (CS) was presented. Shock sensitivity was evaluated following completion of the conditioned fear test as previously described [17].

### Statistical Analyses

In general, analysis of variance (ANOVA) models were used to evaluate the data. Most behavioral data were analyzed using a repeated measures factorial model containing two between-subjects variables (Group and Gender), and one within-subjects variable (e.g., Blocks of Trials). The Huynh-Feldt (H-F) adjustment of alpha levels was used for all within-subjects effects containing more than two levels in order to protect against violations of the sphericity/compound symmetry assumptions underlying the repeated measures ANOVA model. Pairwise comparisons (Bonferroni corrected significance levels) were conducted after significant main effects of Group or significant interactions involving Group across time variables like Blocks of Trials.

## Results

### Neurodegenerative Effects of PCP

**Figure 1:**
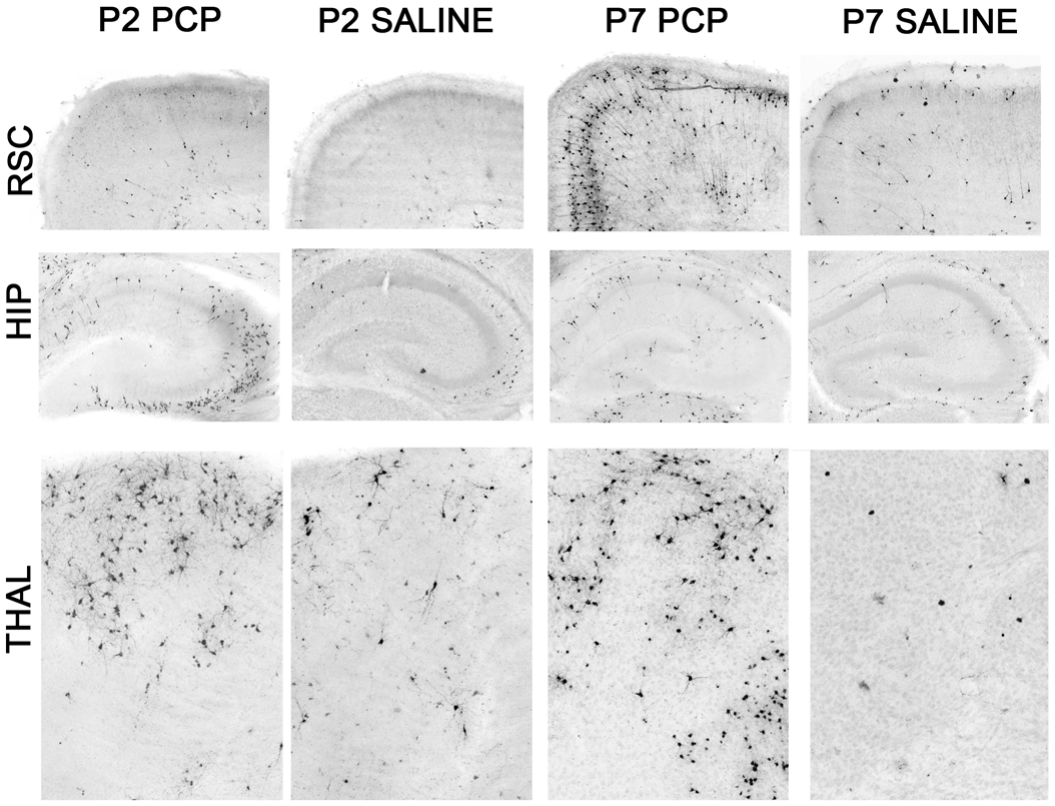
Pattern of neuroapoptosis following PCP treatment in neonatal mice differs depending on whether exposure occurs on P2 or P7. Photomicrographs showing activated caspase3- (AC3-) positive cell profiles indicating neuroapoptotic damage in the retrosplenial cortex (RSC), hippocampus (HIP), OR anterodorsal thalamic nuclei (THAL) in mice that were treated with PCP on either P2 (P2PCP) or P7 (P7PCP) or with saline on the same postnatal days (P2 Saline and P7 Saline, respectively). PCP exposure on P2 produced neuroapoptotic damage to the HIP and THAL regions without affecting the RSC. In contrast, PCP exposure on P7 resulted in severe neuroapoptosis in the RSC and moderate damage to the THAL, while leaving the HIP relatively spared. Images taken at 10× magnification.

**Figure 2:**
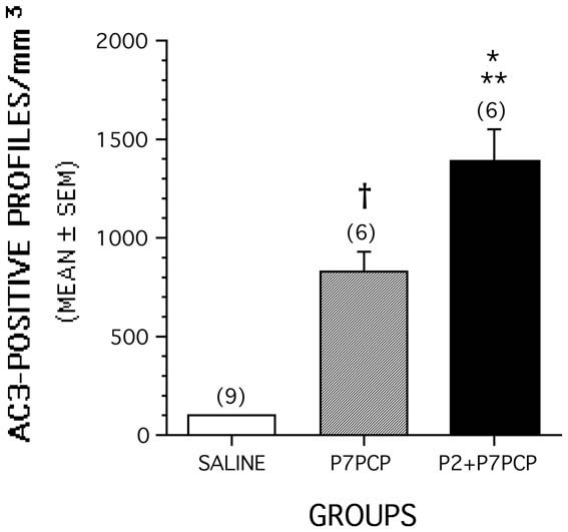
Density of AC3-positive profiles is greater when PCP exposure occurs on P2 and P7 (P2+P7) compared to exposure on P7 alone. Density of AC3-positive cell profiles summed across forebrain regions most sensitive to PCP-induced neuroapoptosis on P7 (retrosplenial cortex, caudate putamen, anterior thalamic nuclei) is significantly (**p = 0.012) greater in mice that were exposed to PCP on P2 and P7 (P2+P7PCP group) compared to that observed in mice that were treated only on P7 (P7PCP). The P7PCP and P2+P7PCP groups each showed significantly greater densities of AC3-positive profiles compared to the saline controls (†*p* = 0.001; *p<0.00005, respectively). Numbers in parentheses indicate sample sizes of each group.

Evaluation of coronal sections stained by AC3-immunohistochemistry revealed that all brains exposed to PCP either at P2, P7 or P2 + P7 had substantially larger numbers of stained profiles in many different regions than were present in the saline control brains. Consistent with a prior report in rats pertaining to neuroapoptosis induced by NMDA antagonists [1], there were some similarities but also major differences in the pattern of neuroapoptosis that occurred following PCP exposure on P2 compared to that observed following exposure on P7. Notable similarities involved dense staining of AC-3 positive profiles in the anterior and laterodorsal nuclei of the thalamus and the caudate/putamen following PCP exposure at both ages. The most prominent differences included a high density of stained profiles in the hippocampus and certain subcortical structures such as the hypothalamus and amygdala, but very few stained profiles in the neocortex in mice exposed to PCP on P2. In contrast, all divisions of the neocortex showed a high density of staining in mice exposed to PCP on P7 with the hippocampus, hypothalamus and amygdala being less prominently affected (**Fig. 1**). On the basis of qualitative comparisons, it appeared, in general, that there were more densely stained AC3-positive profiles in the P2+P7 PCP-treated brains compared to that observed in the P7 alone PCP group. This was confirmed by quantitative counts of stained AC3-positive profiles that were summed across three representative regions that appeared to contain the greatest density of staining (retrosplenial cortex, caudate putamen, anterior thalamic nuclei). Statistical analysis of the quantitative counts demonstrated that the density of AC3-positive profiles was significantly different among the treatment groups [F(2,14) = 34.92, p<0.0001], with subsequent pairwise comparisons showing that the density of profiles was significantly greater in the P2+P7 PCP-treated group compared to levels observed in the P7 PCP-treated mice (p = 0.012). Also, each of the two PCP-treated groups showed significantly greater staining density compared to the saline group (p<0.002) (**Fig. 2**).

### Effects of PCP on Body Weight

**Figure 3:**
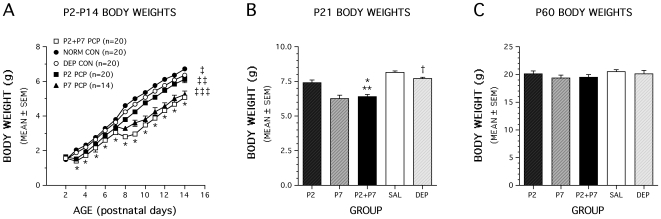
PCP treatment on P2 and P7 (P2+P7) significantly reduces body weights relative to both normal and deprived controls during the early pre-weaning period. (A) An ANOVA on body weights yielded a significant main effect of Group, [F(2,57) = 139.17, p<0.0005], and a significant Group by Postnatal Day interaction, [F(24,684) = 76.07, p<0.000], showing that there were different patterns of body weights in the three groups across P2-P14. Groups differed significantly (*p<0.0005) on each postnatal day except P2. Across postnatal days, the PCP-treated mice weighed significantly less than either the normal (ààp<0.0005) or deprived (àp<0.0005) control groups, and the deprived mice also weighed significantly less than normal controls (àààp = 0.005). (B) The groups also differed significantly in body weights at weaning (P21), (*p<0.0005), and pairwise comparisons indicated that the PCP-treated mice weighed less than either the normal or deprived controls (*, **p<0.0005), and the deprived group weighed less than the normal controls ( p = 0.013). (C) Analysis of absolute body weights at P60 indicated no significant differences. P2 and P7 PCP single treatment groups are included for general comparison.

Animals treated with a single dose of PCP on P2 weighed significantly less than either normal (suckle) (p = 0.009) or deprived controls (p = 0.00001) across postnatal days P2-P14. Overall ANOVA indicated significant differences among groups at P21 [F(2,57) 3.326, p = 0.043], however differences between groups were not significant in post-hoc analysis and by P45 no significant difference were observed among groups. Exposure to a single dose of PCP on P7 also resulted in decreased body weights across postnatal days P7–14 compared to deprived controls (p = 0.00009) and at P21 [F(1,28) = 11.974, p = 0.0017). Body weight analysis at P45 revealed no significant differences between groups. An analysis of body weights from the P2+P7 PCP experiment showed that across postnatal days P2–P14, the PCP-treated mice weighed significantly less than either the normal (suckle) or deprived control groups (p<0.0005) (**Fig. 3A**). In addition, the deprived mice also weighed significantly less than normal controls (p = 0.004) across these postnatal days suggesting that at least some of the decreases in body weights in the PCP-treated mice resulted from the time the pups were denied access to the dam. Analysis of body weights at weaning (P21) showed the same pattern of significant results (**Fig. 3B**). Although there were relatively long-term differences in absolute body weights, an analysis of body weight changes over the P2–P14 period showed that significant losses of body weight in the PCP-treated mice were restricted to the first post-treatment day (data not shown). Moreover, the PCP-treated mice rebounded quickly and actually showed greater body weight gains than either control group by P9–10, an effect that remained until P17–18 (not shown). In spite of the differences in absolute body weights, no obvious developmental abnormalities or differences in general health were observed among the three groups. Analysis of absolute body weights measured at (P60) showed no significant differences among PCP-treated animals and controls (**Fig. 3C**).

### Behavioral Effects of PCP Exposure on P2

Preliminary analyses were conducted between the normal and deprived controls with regard to the data from the cued, place and probe conditions in the water maze and from the conditioned fear task, with the result being that no differences were observed between the two groups on any of the learning and memory measures. As a result, the data from the two groups were combined and ANOVAs were conducted on the P2 PCP-treated mice and the combined control group in an effort to clarify the effects of PCP treatment on learning and memory.

Animals exposed to PCP on P2 did not differ from controls on measures of locomotor activity, sensorimotor function, water maze acquisition at P30, or of the ASR/PPI, contextual fear or auditory cue tests, or on water maze acquisition and retention measured at P75 (data not shown). However, they did show mild retention impairments in water maze testing at P30 as indicated by a decreased number of platform crossings compared to control groups during the first probe trial [F(1,58) = 9.99, p = 0.002]; this difference was no longer significant by the second probe trial conducted at the end of place training (data not shown).

### Behavioral Effects of PCP Exposure on P7

Animals exposed to PCP on P7 did not differ from controls on measures of locomotor activity, sensorimotor function, water maze acquisition or retention at P30 or P75, or of the ASR/PPI. No significant differences between groups were observed on the contextual fear test, or on a test of shock sensitivity. However, mice exposed to PCP on P7 exhibited significantly less freezing in response to the tone during the auditory cue test compared to saline controls [F(1,28) = 4.43, p = 0.02] (data not shown).

### Behavioral Effects of PCP Exposure on P2+P7

Analyses of the normal and deprived control groups again showed no significant differences on any learning and memory measures at any age (P30, P75, P170) so the data from the two groups were combined for statistical analyses conducted on the P2+P7 PCP-treated mice in an effort to simplify the interpretation of the effect of PCP treatment on learning and memory.

Similar to the results from the studies involving PCP treatment on P2 or on P7, there were no significant differences between groups on the early post-weaning 1 hr locomotor activity test including indices of altered emotionality, or on any of the measures from the sensorimotor battery (data not shown). Likewise, there were no significant overall effects observed on any of the variables from ASR/PPI testing conducted on P50 (data not shown).

### PCP Exposure on P2+P7 Produces Severe Spatial Learning and Memory Impairments at P30

**Figure 4:**
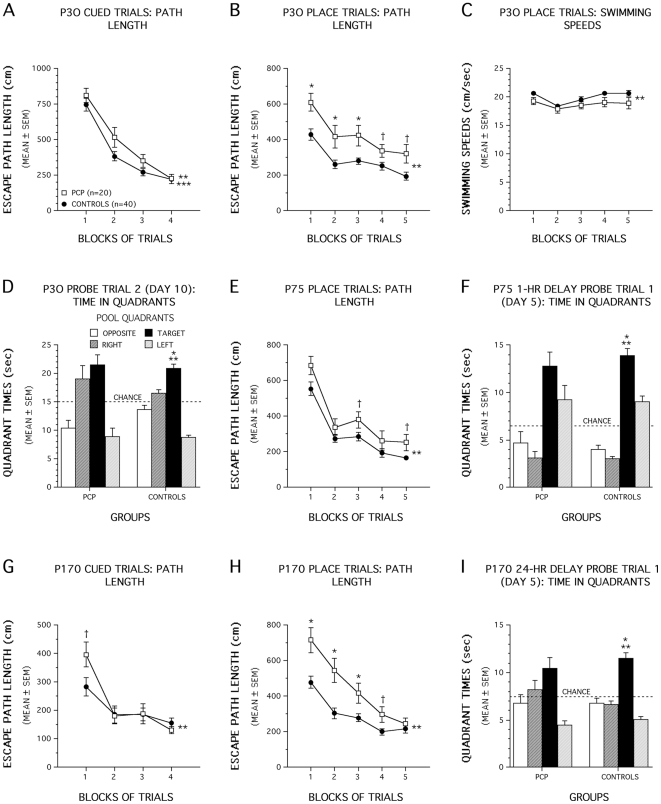
P2+P7 PCP treatment impairs spatial learning and memory in the Morris water maze. (A) An effect of Group (**p = 0.015) and a Group by Gender by Blocks of Trials interaction (***p = 0.017), suggested group differences in path length over trials in the cued condition. However, PCP-treated mice did not differ significantly from control mice on any single block of trials suggesting differences were minimal, and additional contrasts did not indicate meaningful gender effects. (B) PCP-treated animals showed significant acquisition deficits at P30 (**p<0.0005) for blocks 1, 2, and 3, (*p<0.009) while large differences were found for blocks 4 (†p = 0.042) and 5 (†p = 0.011). (C) Swimming speeds of PCP-treated mice were slower than controls during place trials (**p = 0.031), although pairwise comparisons showed no significant difference in any given block of trials. (D) Control mice showed good retention at the end of training (**p<0.0005). Specifically, they showed spatial bias for the target quadrant (*p<0.0005). (E) PCP-treated mice showed acquisition deficits at P75 (**p = 0.008). Differences were greatest for blocks 3 (†p = 0.049), and 5 (†p = 0.033). (F) For the 1-h delay probe trial control mice exhibited spatial bias for the target quadrant, (**p<0.0005; *p<0.007 for target vs each of the other quadrants) (G) A Group by Blocks interaction (**p = 0.010) was observed for the cued trials at P170. Greatest differences occurred during the first block (†p = 0.023, not significant past Bonferroni correction p = 0.0125). (H) PCP-treated mice displayed acquisition deficits at P170 (**p = 0.003), specifically for blocks 1, 2, and 3 (*p<0.007) and for block 4 (†p = 0.018). (I) During the first 24-h delay probe trial control mice showed spatial bias for the target quadrant (**p<0.0005; *p<0.0005 for target vs each of the other quadrants), while PCP-treated mice did not.

A significant main effect of Group, [F(1,56) = 6.30, p = 0.015], and a significant Group by Gender by Blocks of Trials 3-way interaction [F(3,168) = 3.60, p = 0.017], suggested that group performances with regard to path length differed over the blocks of cued trials (**Fig. 4A**) although differential gender effects may have occurred within treatment groups. Importantly, PCP-treated mice did not differ significantly from control mice on any single block of trials suggesting minimal (if any) performance differences between groups. Other contrasts comparing male PCP-treated mice with male controls and female PCP-treated mice with female controls did not produce significant performance differences between the groups, on average across the blocks of trials, suggesting that there were no meaningful effects of PCP treatment as a function of Gender. Clearly, by the last block of cued trials, virtually identical performance levels were observed in the two groups (**Fig. 4A**). Similar, although slightly larger, overall effects were observed for escape latency (data not shown). An ANOVA of swimming speeds over trials did not yield any significant effects involving Group or Gender (data not shown). In summary, small differences may have existed between P2+P7 PCP-treated and control mice during the cued trials resulting in transient performance deficits suggesting that mild non-associative disturbances in the PCP-treated mice (e.g., visual or sensorimotor disturbances, altered motivation) may have briefly affected performance although the two groups eventually performed at approximately equal levels.

In contrast to the small effects of P2+P7 PCP treatment on cued trials performance, PCP-treated animals showed robust and significant acquisition deficits during the place condition of the water maze at P30 (**Fig. 4B**), [Group main effect: F (1,56) = 19.03, p<0.0005], but no other overall effects involving Group or Gender were significant. Subsequent pairwise comparisons showed that differences between PCP-treated and controls were significant beyond Bonferroni correction (p = 0.05/5 = 0.01) for blocks 1, 2, and 3 (p<0.009), while differences were also large for blocks 4 (p = 0.042) and 5 (p = 0.011). The same effects were observed in the analysis of the latency data but were generally greater in magnitude (data not shown). An ANOVA of the swimming speed data (**Fig. 4C**) showed a significant main effect of Group, [F(1,56) = 4.91, p = 0.031], and a significant Gender by Group interaction, [F(1,56) = 11.64, p = 0.001]. However, subsequent pairwise comparisons showed no significant differences beyond Bonferroni correction (p = 0.01) between PCP-treated and control mice for any given block of trials. Additional contrasts indicated that the Gender by Group interaction effect was mostly due to the PCP-treated males swimming significantly slower, on average across trials, compared to the control male mice (p<0.0005), while no differences were observed between the two groups of females (data not shown). Analysis of the data from the first probe trial conducted midway through acquisition (day 5) showed no evidence of spatial bias in either group for the target quadrant and no differences were observed in platform crossings. An ANOVA of the platform crossing data from the second probe trial (day 10), revealed a significant Gender by Group interaction in platform crossings [F (1,56) = 7.08, p = 0.010], but no other overall effects including Group. However, the lack of significant performance differences following comparisons conducted between the two male groups and between the two female groups suggested that there were no meaningful gender-based effects of PCP treatment. The control mice exhibited good retention of the platform location by the second probe trial at the end of training (day 10) when they showed a significant spatial bias for the target quadrant where their search times were significantly greater than those observed in each of the other quadrants (p<0.0005), while the PCP-treated mice showed no such bias for the target quadrant (**Fig. 4D**).

### P2+P7 PCP− treated mice are still impaired on the water maze task during retesting at P75 but show signs of recovery

Retesting the mice on a new hidden platform location at P75 indicated a general reduction in performance differences between the two groups suggesting partial recovery of function on the part of the PCP-treated mice (**Fig. 4E**). Nevertheless, significant main effect of Group, [F (1,56) = 7.69, p = 0.008], showed that, in general, the path lengths of the PCP-treated mice were significantly longer than those of the control group during acquisition of the new platform location. Subsequent comparisons indicated that differences between the groups were greatest for blocks 3 (p = 0.049), and 5 (p = 0.033]. The same pattern of overall effects was found for the latency data (not shown). The ANOVA on the swimming speed data showed a significant Group by Gender interaction, [F(1,56) = 4.84, p = 0.032], although subsequent pairwise comparisons showed no differences between PCP-treated and control males or between PCP-treated and control females indicating that this effect had little meaning with regard to the effect of PCP treatment. Probe trial performance midway through acquisition training (day 5) indicated no significant differences between the groups in terms of platform crossings or spatial bias for the target quadrant during the 24 hr delayed probe. However, although no differences between groups were found for platform crossings in the 1 hr delay probe trial on day 5, the control mice exhibited a significant spatial bias for the target quadrant, (p<0.007 for target vs each of the other quadrants), while the PCP-treated group did not (**Fig. 4F**). Regarding probe trial performance at the end of acquisition (day 10), no significant differences were observed between the two groups for either probe trial in terms of platform crossings, and both groups showed significant spatial bias for the target quadrant (data not shown). In summary, the results from the water maze testing at P75 indicated that the PCP-treated mice still had subtle deficits relative to the control mice during acquisition and retention of the new platform location.

### P2+P7 PCP-treated mice showed robust deficits when retested on the water maze task at P170 in the absence of continued handling and testing

In order to determine whether the apparent recovery of function in PCP-treated mice continued beyond water maze testing at P75, the mice were left unhandled except for cage and bedding changes for 70 days beginning around P100 and then were re-tested at P170. We were particularly interested in determining whether the spatial learning capabilities of the PCP-treated mice would become normalized during testing at a later age. Because of the slight differences observed in cued trial performance at P30, we decided to conduct the cued trials again to determine if any deficits were still demonstrable in the PCP-treated mice. Analysis of the path length data from cued trials testing at P170 provided some weak evidence that the performance of the PCP-treated mice may have been subtly impaired during the cued condition (**Fig. 4G**). For example, a significant Group by Blocks interaction [F(3,168) = 4.08, p = 0.010] was found suggesting different patterns of group performances occurred across blocks of trials. However subsequent pairwise comparisons showed that the greatest differences in path lengths between groups were found during the first block of trials (p = 0.023), although this difference was not significant following Bonferroni correction (p = 0.0125). The same pattern of results was found for the latency data (not shown). An ANOVA of the swimming speed data yielded a significant main effect of Gender, [F(1,56) = 31.04, p<0.0005], a significant Group by Gender interaction, [F(1,56) = 20.37, p<0.0005], and a significant Group by Blocks interaction, [F(3,168) = 2.90, p = 0.036]. Subsequent contrasts showed that these effects were at least partially due to the PCP-treated males being significantly slower swimmers than the PCP-treated females, on average across the blocks of trials (p<0.0005), whereas no gender differences were observed with regard to swimming speeds for the control mice (data not shown). Importantly, however, differences in swimming speeds were not associated with differences in cued learning performance since no gender-based effects were found on these variables. Thus, the cued data from this adult water maze testing was similar to that observed during the earlier testing when the mice were juveniles in that a small, transient impairment may have been present in the PCP-treated mice but only during the initial phase of testing.

Analysis of the P170 place trials data indicated that the PCP-treated mice did not show evidence of continued improvement in spatial learning performance suggesting that further recovery of function did not proceed into later adulthood. Instead, the PCP-treated mice displayed large and significant acquisition deficits during the place condition relative to the control mice that were greater than those observed during the P75 place trials [Group effect: [F (1,56) = 16.77, p<0.0005; Group by Blocks interaction: F (4,224) = 4.41, p = 0.003] (**Fig. 4H**). Subsequent pairwise comparisons showed that PCP-treated mice had significantly longer path lengths compared to the control group for blocks 1, 2, and 3 (p<0.007), while differences were also very large for block 4 (p = 0.018). Similar results were found for the latency data (not shown). An ANOVA of the swimming speed data revealed a Group by Blocks interaction, [F(2,224) = 4.71, p = 0.023], and a Gender by Blocks interaction, [F(4,224) = 5.86, p = 0.010]. Pairwise comparisons showed that the PCP-treated mice had significantly slower swimming speeds compared to controls only during blocks 1 and 2 (p = 0.01). Additional contrasts showed that, similar to the cued trials data, PCP-treated males swam significantly slower than PCP-treated females during blocks 1 through 3 (p<0.0004) while no differences were observed between the male and female control mice (data not shown). Analysis of the probe trial data showed that the PCP-treated mice exhibited inferior retention performance compared to the control group. Specifically, the PCP-treated mice made significantly fewer platform crossings during the first, 24 hr delayed probe trial (p = 0.047) relative to the controls and they never showed significant spatial bias for the target quadrant during any of the four probe trials. In contrast, the control mice showed significant spatial bias for the target quadrant during the first (**Fig. 4I**) and second 24 hr delay probe trials (p<0.0006), but not for either of the 1 hr delay probes. Collectively, the water maze experiments show that the P2+P7 PCP treatment produces long-term, probably permanent, spatial learning and memory impairments although the degree of performance deficits may depend upon stimulation resulting from handling and exposure to other behavioral measures.

### P2+P7 PCP−treated mice showed significant impairments in conditioned fear

**Figure 5:**
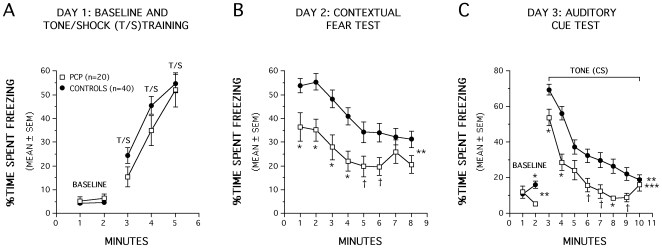
P2+P7 PCP-treated mice showed significant impairments in conditioned fear when tested on P60. (A) Analysis of freezing behavior during the baseline or tone/shock (T/S) pairings on day 1 revealed no significant overall Group effects. (B) PCP-treated mice exhibited significantly lower amounts of freezing compared to the control group when they were returned to the test chamber 24 hrs after training (**p<0.0005). Pairwise comparisons showed that the PCP-treated mice froze significantly less often than controls in response to contextual cues for the first 4 min of the test session (*p<0.0006) while differences were also large for min 5 (†p = 0.040) and 6 (†p = 0.025). (C) An analysis of baseline freezing in response to the altered context on day 3 of auditory cue testing indicated a significant Group by Minutes interaction (**p = 0.001). Subsequent pairwise comparisons showed that this effect was mostly due to differences between the PCP-treated and control mice during min 2 (*p = 0.001). Analysis of freezing in response to the tone presentation yielded a significant main effect of Group (**p<0.0005) and Group by Minutes interaction (***p = 0.048) with pairwise comparisons showing that the PCP-treated mice froze less often than the control group for min 1, 2, and 8 (*p<0.006), while very large differences were also observed for min 6, 7, and 9 (†p<0.025).

The mice were also evaluated on Pavlovian fear conditioning on P60, between the first two water maze tests, to determine whether the P2+P7 PCP-treatment affected other non-spatial forms of learning. Analysis of freezing behavior during the baseline or tone/shock pairings on day 1 revealed no significant effects (**Fig. 5A**) indicating that the two groups exhibited similar levels of baseline freezing upon being introduced to the conditioning chamber and in response to the tone-shock training (CS-US pairings). However, the PCP-treated mice exhibited significantly lower amounts of freezing compared to the control group, [F(1,56) = 14.30, p<0.0005], when they were returned to the test chamber 24 hr after training to evaluate contextual fear conditioning (**Fig. 5B**). The PCP-treated mice froze significantly less often than the controls in response to the contextual cues for each of the first 4 min of the test session (p<0.0006) while differences were also substantial for min 5 (p = 0.040) and 6 (p = 0.025). An analysis of baseline freezing in response to the altered context on day 3 indicated a significant Group by Minutes interaction [F(1,56) = 13.22, p = 0.001], which was mostly due to significant differences between the PCP-treated and control mice during minute 2 (p = 0.001), when the PCP-treated mice exhibited significantly less freezing. Analysis of freezing in response to the tone presentation yielded a significant main effect of Group, [F(1,56) = 14.21, p<0.0005], and a significant Group by Minutes interaction, [F(7,392) = 2.19, p = 0.048] showing that the PCP-treated mice froze less than the controls but the magnitude of the differences was time dependent. Specifically, the PCP-treated mice froze significantly less often than the control group for minutes 1, 2, and 8 (p<0.006), while very large differences were also observed for minutes 6, 7, and 9 (p<0.025) (**Fig. 5C**). However, since we did not include additional control groups for the auditory cue condition, we cannot determine whether impaired performance on this task represents deficits in actual conditioning or in other behavioral processes such as sensitization. Thus, in summary, the PCP-treated group showed impaired contextual fear conditioning and performance deficits during the auditory cue test relative to the control mice, although differences in baseline freezing in response to the altered context may have contributed to the latter effect.

## Discussion

Our findings indicate that exposing mice to PCP on two postnatal days (P2+P7) results in severe, long-term impairment in spatial learning and memory, and in contextual fear conditioning, whereas mouse pups exposed to PCP on only a single postnatal day (either P2 or P7) sustained only minor behavioral disturbances. The P2+P7 group was subjected to Morris water maze testing at 3 ages (P30, P75, P170). Between the P30 and P75 testing, the mice were extensively handled and subjected to frequent behavioral testing, but between P75 and P170 handling was intentionally kept to a minimum. The learning/memory deficits, which were very severe at P30, remained quite severe at P170, but were less severe at P75. We propose that the improved test performance at P75 reflects the frequency of handling and repeated testing in the interval between P30 and P75, and the poor performance at P170 confirms the permanency and severity of neurocognitive impairments resulting from P2+P7 PCP exposure in infancy. In a prior study we reported that exposure of infant mice to alcohol caused severe learning/memory deficits at P30 followed by an apparent partial recovery of function when tested at P75. The present results suggest that frequent stimulation and familiarity of continued behavioral testing provides some remediation in mice treated with either alcohol or PCP in infancy, but it may not lead to genuine neural repair or complete recovery of cognitive function.

Our findings support and extend prior work by Wang et al., [8], [9], who reported that exposing female rat pups to PCP on three postnatal days (P7+P9+P11) slowed acquisition of a delayed spatial alternation task when testing began on P42. In the present study we have demonstrated that robust spatial learning and memory deficits occur in mice that were subjected to two PCP exposures (i.e., on P2+P7) but not as a result of a single exposure on P2 or P7, and that these impairments are likely to be permanent in nature and encompass non-spatial forms of learning as well. In addition, Wang and colleagues focused on the effects of PCP exposure that occurred later during the developmental period of synaptogenesis (P7, 9 and 11) while our study examined the effects of PCP-induced neuroapoptosis at earlier ages (P2 and 7) to determine the different patterns of neuroapoptosis that occur following single or double dosing regimens during this time.

Our findings confirm earlier observations that the pattern of neurodegeneration induced in the developing brain by an NMDA antagonist drug varies depending on the age at time of exposure [5], [18]. PCP exposure on P2 caused prominent AC3 staining in the hippocampus and certain subcortical regions (e.g., hypothalamus and amygdala), but very limited staining in neocortex. In contrast, PCP exposure on P7 resulted in dense AC3 staining in all divisions of the neocortex, while staining in the hippocampus, hypothalamus and amygdala was much less prominent. Dense AC3 staining was found in the anterior and laterodorsal nuclei of the thalamus and the caudate/putamen following PCP exposure on each of these postnatal days. Neither the P2 nor P7 pattern was associated with severe learning and memory impairment, but the P2+P7 pattern was. Quantitative counts on P7 assessing the density of AC3-positive profiles in three severely affected brain regions (retrosplenial cortex, caudate/putamen, anterior thalamic nuclei) revealed that the damage was more severe in mice treated with PCP on P2+P7 than those treated only on P7. Because cells dying by apoptosis are no longer AC3 positive approximately 24 hours after drug exposure, the increased AC3 staining in the P2+P7 animals compared to the P7 animals cannot be reflective of damage that is still visible from the P2 exposure. Rather the increased staining in the P2+P7 animals indicates that PCP exposure at P2 has produced alterations in the brain that result in it being more susceptible to the apoptotic effects of PCP at P7.

Traditionally, spatial learning and memory deficits have been linked to hippocampal dysfunction. However, there is substantial evidence that an extended extra-hippocampal circuit plays a role in mediating allocentric (landmark-based) spatial learning and memory functions such as those evaluated in rodents with the water navigation task [19], [20]. This circuit includes, in addition to the hippocampal formation, the retrosplenial cortex, anterior thalamic nuclei and mammillary bodies [19]. In the P2+P7 group all of these regions were involved except for the mammillary bodies. In a prior study [7] we showed that alcohol damages all of these regions, including the mammillary bodies, but the latter occurs on a delayed basis (16–24 hrs) and would not have been detected in the present study (histological evaluation at 10 hrs). In addition, neuroapoptosis in the caudate/putamen may amplify these effects since it has been shown to be involved in incremental learning involving stimulus-response associations, and there is evidence implicating the medial dorsal striatum in place learning in the water maze [21].

Nutritional status may have played a contributory role to the cognitive deficits observed in the P2+P7 PCP-treated mice, although the evidence suggests otherwise. For example, transient weight loss in the period immediately following treatment occurred in all PCP-treated groups of mice, with absolute body weights remaining lower than control mice until early adulthood. However, PCP-treated mice showed normal weight gains within two days post-treatment suggesting a quick resumption in normal growth rates in the drug-treated mice. Evidence that nutritional deficiencies had little or no impact on the behavior of P2+P7 PCP mice come from several sources. For example, the P2+P7 PCP-treated mice did not exhibit impaired performance during the 1 h locomotor activity test, either in terms of general activity or emotionality variables relative to the control mice. The P2+P7 PCP mice also did not display performance deficits on any of the measures within the sensorimotor battery or in terms of ASR/PPI responsivity. In addition, the groups of mice that were subjected to single exposures of PCP on either P2 or P7 had significantly decreased body weights for similar periods of time but showed little or no disturbances in learning and memory performance in the above-mentioned behavioral tests. Specifically, animals treated with a single dose of PCP at P7 weighed the same as, if not slightly less, than P2+P7 PCP treated mice at P21 without showing severe cognitive impairment. In addition, the deprived control mice weighed significantly less than the normal suckle control group yet no differences were found between these two groups on any of the behavioral measures.

Our finding that exposure of infant mice to PCP on two occasions causes much more severe neurobehavioral impairments than PCP exposure on only one occasion, may have relevance in a public health context. There is mounting evidence that anesthetic drugs, including the NMDA antagonist, ketamine, trigger neuroapoptosis in the brains of infant rodents [22]–[25], and monkeys [26]–[28], and there is preliminary evidence potentially linking anesthesia exposure in infancy with long-term neurobehavioral deficits in humans [29]–[31]. In one recent human study [30] it was found that the risk of neurobehavioral disturbances was significantly increased if the duration of anesthesia exposure was greater than 120 minutes or if anesthesia exposure occurred more than one time. This is consistent with our finding in infant mice that PCP, a dissociative anesthetic with NMDA antagonist properties, causes much more severe neurobehavioral impairments if exposure occurs on more than one occasion.
